# Active surveillance with telemedicine in patients on anticoagulants during the national lockdown (COVID-19 phase) and comparison with pre-COVID-19 phase

**DOI:** 10.1186/s43044-020-00105-w

**Published:** 2020-10-16

**Authors:** Gurbhej Singh, Samir Kapoor, Vasu Bansal, Mehtab Grewal, Bhupinder Singh, Abhishek Goyal, Rohit Tandon, Shibba Takkar Chhabra, Naved Aslam, Gurpreet Singh Wander, Bishav Mohan

**Affiliations:** 1 Department of Cardiology, Hero DMC Heart Institute, Unit of Dayanand Medical College and Hospital, Ludhiana, India; 2 Department of Cardiothoracic and Vascular Surgery, Hero DMC Heart Institute, Unit of Dayanand Medical College and Hospital, Ludhiana, India

**Keywords:** Telemedicine, Oral-anticoagulation, COVID-19

## Abstract

**Background:**

The COVID-19 pandemic brought restriction to the movement of people due to the implementation of lockdown across various regions around the world. In India, most of the patients belong to rural areas and hence were unable to come for a follow-up visit. Hence, we reached out to patients on oral anticoagulation using telemedicine with aim of communicating with the patient concerning drug compliance, titration of dose of anticoagulation, health education, and identification of high-risk patients needing referral to the nearest health facility/our institute. This study was conducted at the Hero DMC heart institute (a tertiary care center for cardiac diseases). The study design is cross-sectional and involves a comparison of the pre-COVID-19 phase with the COVID-19 phase. We asked a five-component (Likert scale) questionnaire from patients for satisfaction after the consult. All symptoms, need for hospitalization and clinical events were recorded. The events were compared in both groups.

**Results:**

We contacted 628 patients through telemedicine and 600 patients gave consent for participation in the study. For comparison, we analyzed data of 614 patients in the pre-COVID-19 phase. The mean age during the pre-COVID-19 phase was 55.27 + 17.09 years and the COVID-19 phase was 56.97 + 15.09 years with males more than females in both groups. There was no significant difference in the number of patients on oral anticoagulants and novel oral anticoagulants (NOAC). However, there were higher number of  patients on antiplatelets in the pre-COVID phase (*p* value0.01). 37% in the pre-COVID-19 phase and 40.31 % in the COVID-19 phase were noted to have out of target range INR (International normalized ratio). There was no difference in the number of bleeding or thromboembolic events seen. Patient response as assessed by a questionnaire (Likert scale) showed that >75% of patients were satisfied.

**Conclusion:**

Through telemedicine, we were able to approach our patients on oral anticoagulation and achieved titration of anti-coagulation, and health education similar to pre-COVID-19 times. During pandemics, telemedicine offers a promising option for patient management with chronic cardiac conditions. It also provides us an opportunity for the management of patients on oral anticoagulation involving titration of drug dosages (anti-coagulation), identification of high-risk patients, and health education.

## Background

Although a novel coronavirus outbreak was noted in December 2019, COVID-19 (coronavirus disease-19) was labeled as a pandemic by the World Health Organization on 11 March 2020. That meant the necessary precautions to be followed by countries around the world to control the spread of infection. In India with a huge population and limited health resources, there was an urgent need to slow down the transmission. The earliest step implemented was a lockdown announcement by the government. This meant restricted movement of people with the shutdown of services except for the essential ones. One of the major tasks for a tertiary care center in northern India catering to the health needs of millions of patient population was to outreach the patients through telemedicine [1, 2]. Telemedicine has been used previously and shown to be effective in chronic neurological conditions, renal conditions, malignancies, and rehabilitation services [3, 4]. Hence, we started telephonic, social media, and e-consultations for our patients. We, as a cardiology unit also started reaching out to patients to know about well-being and medical needs. Whereas cardiovascular disease (CVD) is a risk factor and does seem to play a role in the morbidity and mortality of COVID-19, this viral infection itself has potential mechanisms to cause cardiac and vascular injury [5]. India, being a state of diverse educational, medical, and living conditions, self-monitoring [6] is not easy to achieve especially in rural populations. We took this opportunity to educate our cardiac patient population about the spread of COVID-19 and precautions to be followed. One of our large strata of patients consisted of patients on oral anticoagulation. In this study, we aimed to compare the effectiveness of the management of patients on oral anticoagulants through telemedicine during the COVID-19 phase versus the pre-COVID-19 phase.

## Methods

**Fig. 1:**
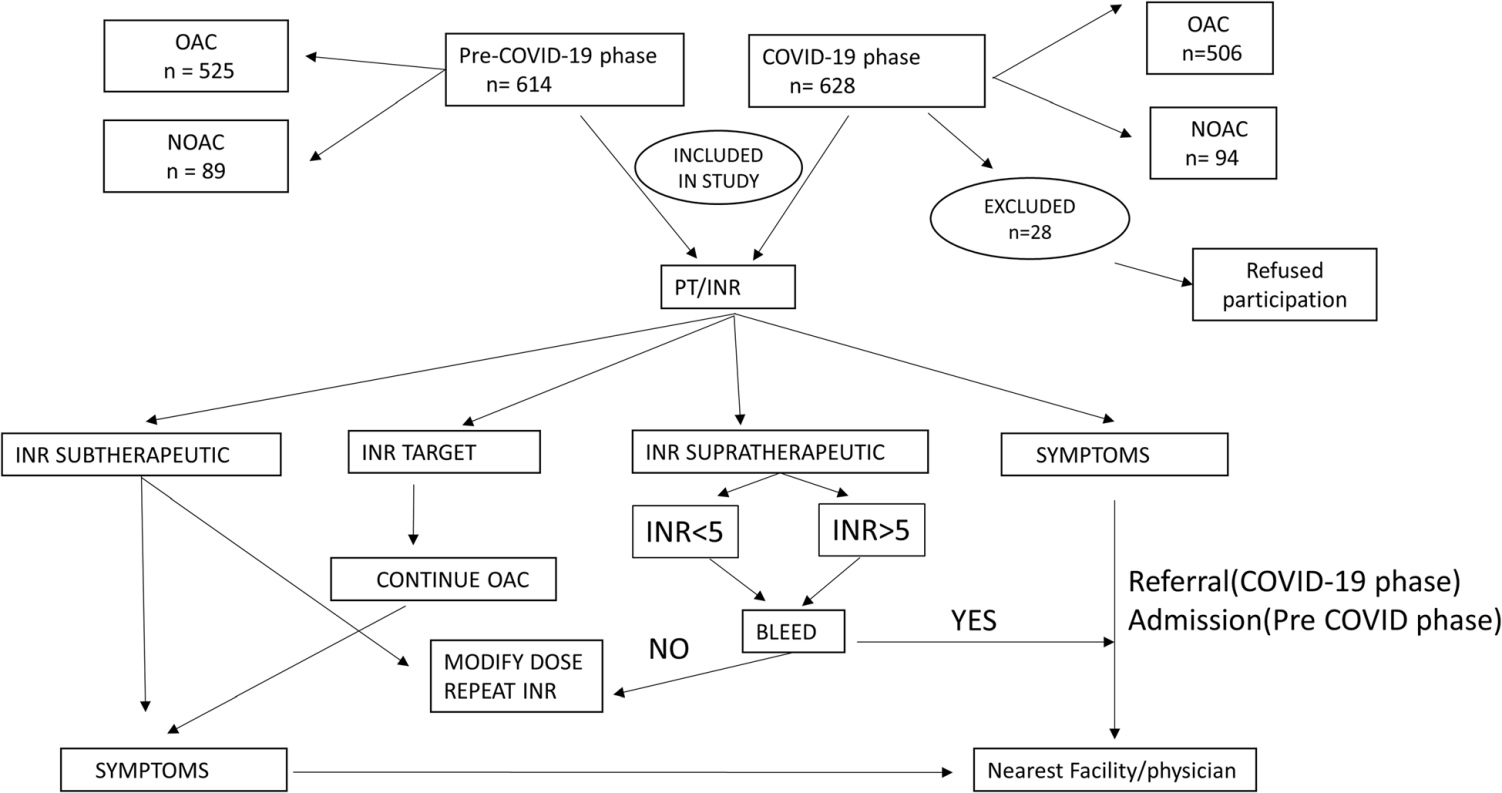
Flow chart showing the methodology of the study (INR, international normalized ratio)

This study was conducted at the Hero DMC Heart Institute (a tertiary care center for cardiac diseases). The study design is cross sectional, and the pre-COVID-19 phase was taken as November-December 2019, whereas the COVID-19 phase was considered as March-April 2020. The data from all outpatient visits were taken from hospital records. All teleconsultations were done strictly under the regulations set by the government and regulatory authorities. All the patients who were scheduled for appointments in March-April 2020 were contacted. Using patient records, a team of 5 (3 nurses and 2 interns) called 628 patients who had been started on anticoagulants. We were able to contact 600 patients for whom prothrombin time (PT) and international normalized ratio (INR) was done near their respective homes. The help of a video consult was taken if patients and relatives wanted. The patients included both, those who had been admitted and those who had visited the OPD. The target INR was according to the current guidelines for respective indications. They were asked if they were experiencing any symptoms presently, in which case they were referred to a cardiac consultant. If they were presently asymptomatic, an indication for which the anticoagulant was started, the drug, dose, the time, and value of the last INR were recorded. In case the last INR value was outside the target, the patients were referred for cardiac consultation. All bleeding events (related to anticoagulation) and thromboembolic episodes were noted. Any referrals to the nearest physician/cardiologist were noted (Fig. 1). Besides, to assess the response to the telemedicine service, we asked all patients to grade the teleconsultation based on five questions (Likert scale) [7] as given in Fig. 3. This questionnaire was sent to their e-mail/telephonic social media account and they were required to reply in numbers to each question. Further, for patients not having access to these modalities, verbal answers were recorded through at the end of the telephonic consult.

### Statistics

Records were analyzed from both computer and paper formats. Descriptive summaries are presented as frequencies and percentages for categorical data, and as mean and standard deviations or range for continuous variables. We compared categorical variables using Fisher’s exact test/*χ*^2^ test and continuous variables using Student’s *t* test. A *p* value of less than 0.05 is considered significant. All statistical analyses are performed using the SPSS statistical software package (release 20.0, SPSS Inc.; Chicago, IL).

## Results

**Fig. 2:**
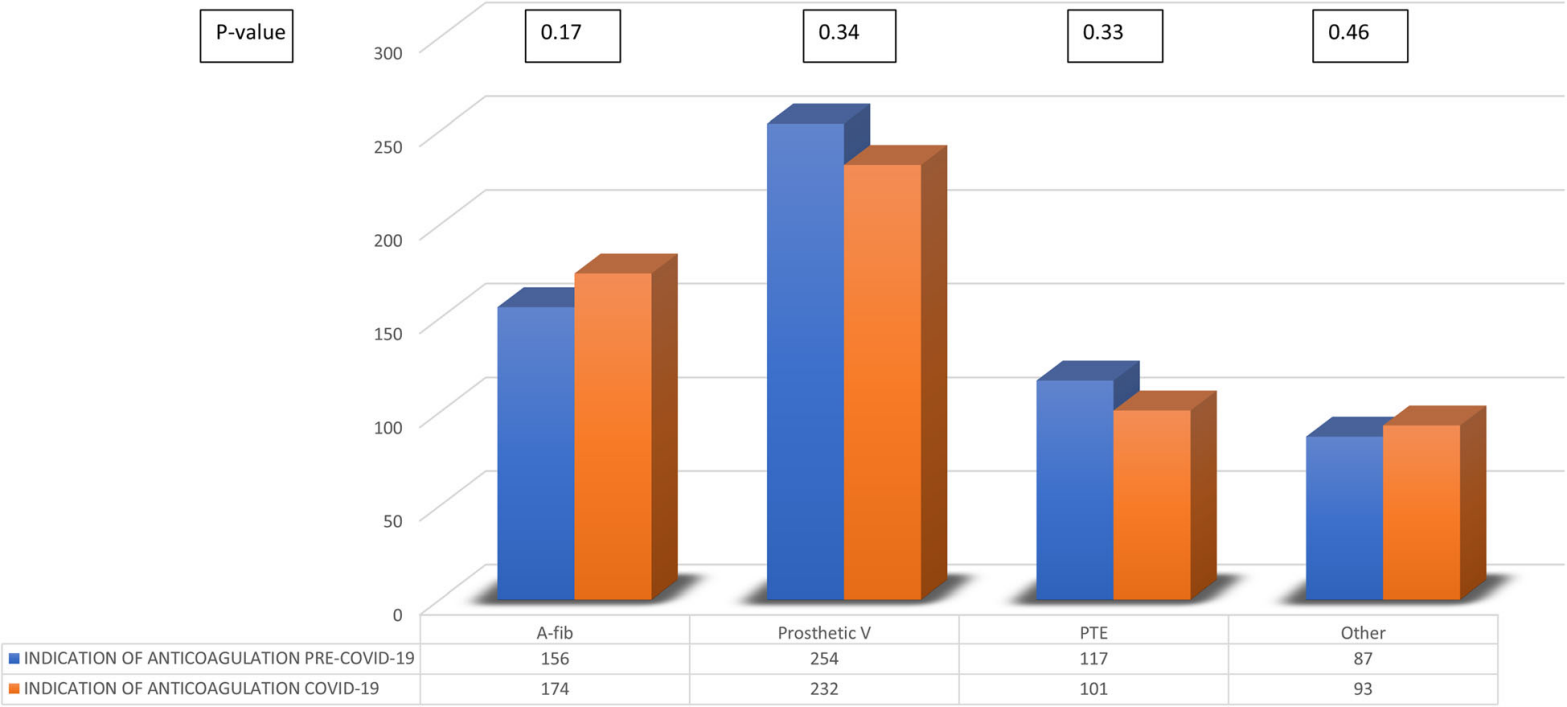
Comparison of indication of anticoagulation in pre-COVID-19 phase versus COVID-19 phase (Afib, atrial fibrillation, PV, prosthetic valve, PTE, pulmonary thrombo-embolism)

A total number of 600 patients were contacted during the Covid-19 lockdown period. Retrospective data of 614 patients were analyzed during the pre-COVID phase. The majority of patients were males. Sixty-four percent of patients were from rural areas. Both groups had a similar number of patients with hypertension, diabetes, and coronary artery disease; however, the number of patients on antiplatelets was higher during the pre-COVID-19 phase as compared to the COVID-19 phase. During both phases, more patients were on warfarin and nicoumalone as compared to the newer anticoagulants (Table 1, Fig. 2).

**Table 1:** Baseline characteristics of patients in COVID-19 phase and pre-COVID-19 phase

Clinical characteristic	Pre-COVID phase	COVID phase	*p* value
Patient (*n*)	614	600	
Age (in years) + SD	55.27 ± 17.09	56.97 ± 15.09	0.56
Sex (M/F)	402/212	378/222	0.37
Rural (*n*, %)	387 (63.03)	398 (66.34)	0.23
Urban (*n*, %)	227 (36.97)	202 (33.67)	
HTN (*n*, %)	238 (38.76)	218 (36.33)	0.40
Diabetes (*n*, %)	213 (34.6)	189 (31.5)	0.24
CKD (*n*,%)	73 (11.88)	54 (9)	0.11
CAD (*n*, %)	205 (33.38)	189 (31.5)	0.50
***Indication of OAC***			
Atrial fibrillation (*n*, %)	156 (25.4)	174 (29)	0.17
Prosthetic valve (*n*, %)	254 (41.3)	232 (38.7)	0.34
Pulmonary thromboembolism (*n*, %)	117 (19.1)	101 (16.9)	0.33
Other (*n*, %)	87 (14.16)	93 (15.5)	0.46
***Drugs***			
OAC (*n*, %)	525 (86.6)	506 (84.4)	0.57
NOAC (*n*, %)	89 (14.4)	94 (15.6)	0.57
Antiplatelets (*n*, %)	267 (43.48)	218 (36.33)	0.01

*OAC* oral anticoagulants-warfarin and nicoumalone, *HTN* hypertension, *CKD* chronic kidney disease, *CAD* coronary artery disease

We found that a significantly higher number of patients had symptoms during the pre-COVID-19 (16.7%). These patients came with symptoms during the pre-COVID-19 phase for out-patient visits and most of them had complaints of worsening of dyspnea (10.6%) followed by palpitations (9.6%).

During the COVID-19 phase, 9.6% of patients on teleconsultation told that they are symptomatic with the majority complaining of worsening dyspnea (6.8%). All the symptomatic patients were asked to visit the nearest health facility for their respective complaints (Table 2).

**Table 2:** Symptomatic patients in pre-COVID-19 versus COVID-19 phase

	Pre COVID-19 (*n* = 614)	COVID-19 (*n* = 600)	*p* value
Symptomatic	103 (16.7)	58 (9.6)	0.003
Referral (*n*)	NA	58 (9.6)	
Deranged INR	195 (37.14)	204 (40.31)	0.30
Subtherapeutic	137 (26.1)	157 (31)	0.08
Supratherapeutic INR < 5	58 (11.04)	47 (9.2)	0.35
Supratherapeutic INR > 5	23 (4.3)	18 (3.5)	0.52
***Clinical events***			
Hospitalization	29 (4.7)	19 (3.1)	0.18
Major bleeding	7 (1.1)	3 (0.5)	0.34
Minor bleeding	4 (0.6)	5 (0.8)	0.75
Thromboembolic episode	15 (2.4)	10 (1.6)	0.42
CVA/TIA	11 (1.7)	7 (1.1)	0.77
Prosthetic valve thrombosis	4 (0.6)	3 (0.5)	1.00

*INR* international normalized ratio, *CVA* cerebrovascular accident, *TIA* transient ischemic attack

In addition, all patients who had INR of more than 5 were asked to visit a physician/cardiologist if convenient or to withhold anticoagulation and repeat PT/INR after 24-48 h. Patients with deranged PT/INR were similar in both the groups (pre-COVID-19—37.14% versus COVID-19 phase—40.31%) with most of the patients having subtherapeutic PT/INR. Patients requiring hospitalization were 29 (4.7%) in the pre-COVID-19 phase versus 19 (3.1%) during the COVID-19 phase. Most of the hospitalizations during the pre-COVID-19 phase were for heart failure (*n* = 15) followed by thromboembolic complications. However, during the COVID-19 phase, most of the admissions were for thromboembolic episodes and major bleeding. Five patients refused admission during the COVID-19 phase. The events did not differ between the two groups significantly.

**Fig. 3:**
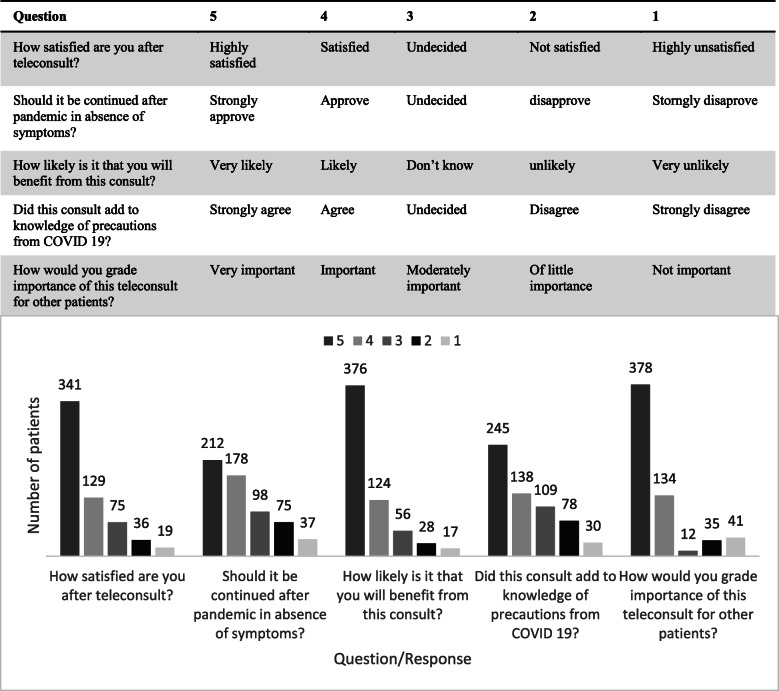
Various responses to questions in the questionnaire as given by patients during telemedicine consult (COVID-19, coronavirus disease-19)

The results of the simple questionnaire are summarized in Fig. 3. Overall, more than 75% of patients were satisfied with this form of consultation (56.8% highly satisfied and 21.5% satisfied). Despite this, 35% of the patients wanted that physical consultation with the physician should be started after the pandemic ends.

Three patients were evaluated for worsening heart failure and were found to have increased valve gradients. Two patients had bi-leaflet mechanical mitral valves and one patient had a tilting disc aortic mechanical valve.

Two patients had PT/INR higher than targets and one had subtherapeutic INR (stopped medicine) and fluoroscopy was used to confirm the diagnosis. Two patients were thrombolysed with fibrinolytic agents and one patient died due to complications of fibrinolytic agents.

## Discussion

To the best of our knowledge, this study is the first in our country comparing pre-COVID-19 versus COVID-19 phase surveillance in patients on oral anticoagulants. It is well understood that suboptimal INR values are associated with complications of stroke and mortality [8]. Through telemedicine, we could reach out to these specific strata of patients requiring dedicated follow-up for titration of INR. Thus telemedicine could be used to avoid travel of these high-risk patients out of their homes which indeed helped better implementation of lockdown. This approach avoided travel, exposure to both patients and relatives accompanying patients, and at the same time achieved continuous medical care to the patient. Telemedicine has been used previously for the management of oral-anticoagulation in a study by C. Gardiner et al. in 2006 [9–11]. Through telemedicine, we were able to identify the patients who were high risk based on the symptoms, medicine compliance, availability of medicines, and laboratory investigations. In this study, we arranged for referral of all patients who had symptoms or any complication related to anticoagulation therapy. There was no significant difference noted in the events during the pre-COVID-19 and the COVID-19 phase. In a study done by Menendez-Jandula et al. [12], self-monitoring of PT/INR was found to be comparable to the dedicated anticoagulation clinics; however, this was not applicable for all patients. Most of the previous studies have been done on patients on long term anticoagulation; in our study, we had 18% of patients who were started on anticoagulation within the last 6 months. Most of our patients on follow-up are from rural areas with different educational levels. Hence, self-monitoring for this subset of patients who live in rural areas does not look like a promising option. Flodgren et al. [13] concluded from a meta-analysis of 93 trials that telemedicine success depends on multiple factors like patient population, nature of the disease, severity of the disease, type of the intervention, and health care provider and system. We were able to identify the patients with bleeding events and stroke, and we arranged for a referral to our institute/nearest tertiary care centers. We could demonstrate the feasibility and success of teleconsultation of all patients on anticoagulation. Additionally, patients were satisfied with this teleconsultation during this unprecedented lockdown period. This gives us an insight into the positive perception of patients and their families during difficult times when social distancing is needed and telephonic consult can avoid exposure of cardiac patients from a physical visit. Further, it allowed us to connect to families of patients for health education related to COVID-19, precautions to be followed, and also communicating the health advisories as issued by the government at the time of the study. Another important benefit of this model of patient care is easy access to a specialist when he is not available in person. In our study, for symptomatic patients, the patients were called by a cardiologist/physician to guide them further. Although telemedicine cannot replace the physical consultation during this pandemic period, multiple benefits could be achieved both for the patient, their families, and for the health care/other person associated with the COVID-19 duties to implement the regulations issued by the government while at the same time identifying the patients needing a hospital visit. Our study is a single-center study comparing the patients on oral anticoagulation at two different times and using different methodologies. It is a non-randomized design and involves selection bias. Another limitation is that PT/INR was done at different laboratories which may have affected the results.

## Conclusion

We conclude that telemedicine is an excellent modality allowing for outreach to patients, health education, and early identification of patients requiring a hospital visit and hospitalization, at the same time, avoiding exposure to patients and their family members, health care staff, and persons involved in the COVID-19 duties.

## Data Availability

The datasets used and/or analyzed during the current study available from the corresponding author on reasonable request.

## Abbreviations

COVID-19: Coronavirus disease-19
OAC: Oral anti-coagulation
NOAC: Novel oral anticoagulants
PT: Prothrombin time
INR: International normalized ratio
CVD: Cardiovascular disease
